# Strongyloides hyperinfection syndrome precipitated by immunosuppressive therapy for rheumatoid arthritis and COVID-19 pneumonia

**DOI:** 10.1186/s40794-023-00201-0

**Published:** 2023-10-05

**Authors:** Hasan Hamze, Teresa Tai, David Harris

**Affiliations:** 1 Department of Pathology and Laboratory Medicine, University of British Columbia, 2211 Wesbrook Mall, Vancouver, BC V6T 1Z7 Canada; 2 Department of Medicine, University of British Columbia, 2775 Laurel Street, Vancouver, BC V5Z 1M9 Canada; 3 Department of Medicine, Division of Infectious Diseases, BC Centre for Excellence in HIV/AIDS (BC-CfE), St. Paul’s Hospital, 1081 Burrard Street, Vancouver, BC V6Z 1Y6 Canada

**Keywords:** Strongyloidiasis, Hyperinfection syndrome, Disseminated strongyloides, Tropical medicine, COVID-19

## Abstract

The COVID-19 pandemic has posed clinical and public health challenges worldwide. The use of corticosteroids has become an evidence-based practice to reduce the hyperinflammatory process involved in severe COVID-19 disease. However, this can result in the reactivation of parasitic infestations, even with a short course. We report the case of a 64-year-old Cuban born patient who passed away from *S. stercoralis* hyperinfection syndrome following treatment with dexamethasone for severe COVID-19 disease on a background of prolonged immunosuppression for rheumatoid arthritis. Clinicians should be aware of the risk of strongyloidiasis as a complication of the treatment for severe COVID-19 and other immunosuppressive therapies. We recommend empiric Strongyloides treatment for those who are from, or who have accumulated risk by travelling to endemic areas, and are being treated with corticosteroids for severe COVID-19 disease.

## Introduction

The emergence of SARS-CoV-2 and its rapid spread throughout the world is a major global infectious disease concern [1]. Much research has been conducted to identify the molecular pathways leading to alveolar damage in severe Coronavirus disease 2019 (COVID-19) and targets for management [2]. This research has demonstrated an important role of immune system hyperinflammatory response, especially as it relates to alveolar destruction and organ failure, and forms the rationale for use of corticosteroid therapy in severely ill patients [3]. Since the RECOVERY trial and similar studies have been published, the use of corticosteroids in patients with COVID-19 requiring oxygen therapy has become the standard of practice for severe COVID-19 disease [4]. While this offers a victory for the management of severely ill COVID-19 patients, it comes at a cost to some. Immunosuppressive medications can precipitate reactivation, recrudescence, and the dissemination of subclinical infections, such as strongyloidiasis.

**Fig. 1:**
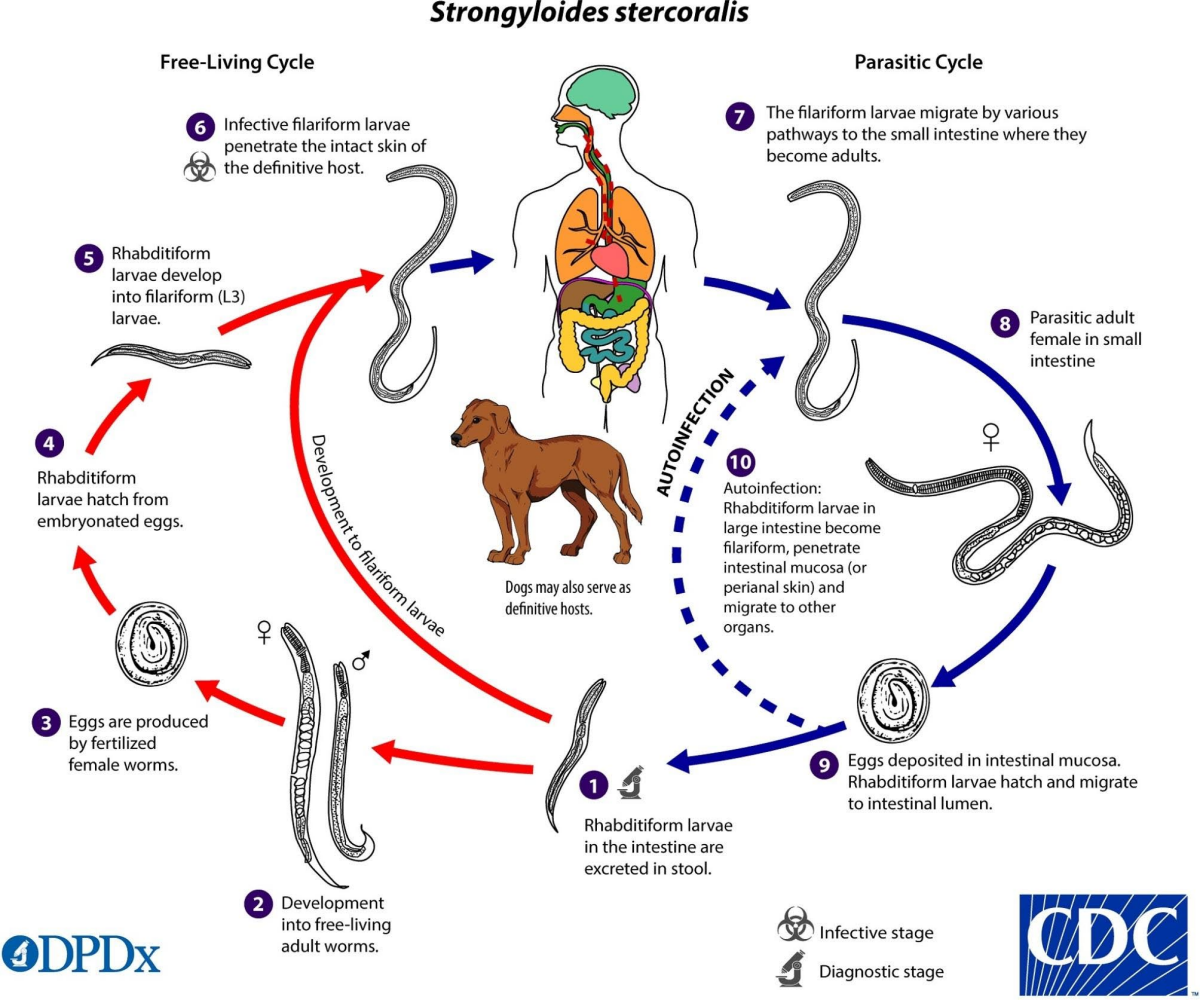
*Strongyloides stercoralis* life cycle (source: CDC)

Strongyloidiasis is a parasitic disease widely distributed in tropical and subtropical regions, with a prevalence of up to 40% in Sub-Saharan Africa, South America, and South East Asia [5]. It is mainly caused by *Strongyloides stercoralis*, an intestinal helminth that spreads primarily through contact with contaminated soil containing the larvae. *S. stercoralis* is distinguished from most other intestinal parasites by its ability to re-infect the host through the wall of the gastrointestinal tract, a phenomenon called autoinfection (Fig. 1) [6].

This autoinfection cycle means humans are infected for life unless treated. *S. stercoralis* infection is often mild or asymptomatic, with up to 64% of patients having no symptoms [7].

Acute Strongyloides infection may cause a cutaneous reaction as the larvae penetrate the skin which has been in contact with soil, most commonly on the foot, but can occur on other areas of skin, or may go unnoticed. If symptoms are appreciated, larvae penetration causes temporary serpiginous or urticarial tracts. Here, the larvae migrate intradermally with a migration speed up to 5–15 cm per hour causing the sign known as larva currens, differentiated from other hookworm infections by its speed of dermal migration. The larvae then penetrate deeper tissues and eventually establish the chronic autoinfection cycle in the host gastrointestinal tract. Larva currens may also occur in chronic infection around the perianal area and upper thighs when the autoinfection occurs in this area. During initial migration to the gastrointestinal tract through the lungs or in cases where larvae migrate out of the gastrointestinal tract to the lungs, a patient may experience respiratory symptoms, such as a dry cough or wheeze, and even pneumonitis with fever. Loffler’s syndrome characterized by fever, wheeze, dyspnea, pulmonary opacifications on chest radiographs, and peripheral eosinophilia, can also occur. When in the gastrointestinal tract, the *S. stercoralis* may not cause any symptoms or may cause varying degrees of anorexia, nausea, heartburn, prandial epigastric pain, and/or diarrhea in up to 23% of patients [6, 7].

Little is known about the relationship between COVID-19 and *Strongyloides* co-infection. Investigating this relationship is critical because many patients with COVID-19 disease receive immunosuppressive treatment. We report a case of fatal sepsis secondary *to Strongyloides* hyperinfection syndrome (SHS) with disseminated strongyloidiasis in a patient who received immunosuppressive treatment for COVID-19 on a background of prolonged immunosuppression for rheumatoid arthritis.

## Case report

A retired 64-year old male construction worker who immigrated from Cuba to Canada over 20 years ago presented to the emergency room with a few-weeks history of acute on chronic epigastric pain, diarrhea, decreased appetite, and general malaise. The patient had no other infectious symptoms and was admitted for possible gastroenteritis.

His past health history included rheumatoid arthritis, for which he had been on methotrexate 10 mg subcutaneously once weekly, hydroxychloroquine 200 mg orally once daily, and sulfasalazine 500 mg orally twice daily for 6 years.

Two days after admission, he became febrile and developed respiratory distress requiring 5 L of oxygen. His chest x-ray demonstrated diffuse interstitial and airspace opacities consistent with interstitial pulmonary edema or infection, and his nasopharyngeal swab was positive for SARS-CoV-2. Given his increasing oxygen requirements, he was transferred to the Intensive Care Unit for respiratory support. He was started on dexamethasone 6 mg IV daily for COVID-19 disease, as well as ceftriaxone and azithromycin empirically for presumptive community acquired bacterial pneumonia. Following that, he developed hypoxemic respiratory failure and required intubation.

On day 6 post ICU admission, a stool ova and parasites (O&P) test that was submitted two days after admission resulted positive for *S. stercoralis* larvae. *Strongyloides* serology (using EUROIMMUN ELISA kits) was also positive at an optical density of 3.335 (cut off is > 1.1). By the time the Infectious Diseases service were consulted, he was on Day 6 of dexamethasone and still intubated requiring 100% FiO2 and haemodynamic support. He was febrile at 38.5 degrees Celsius (101.3 fahrenheit) and had persistent diarrhea. His abdomen was non-tender and there were no new rashes.

His WBC count was 3.1 × 10^9^/L with an eosinophil count of < 0.1 × 10^9^/L on presentation. Blood cultures, stool culture, C. difficile, HIV, and HTLV-1/2 testing were negative. A review of his pre-admission lab work demonstrated a peripheral eosinophilia of 0.9 × 10^9^/L and 0.7 × 10^9^/L six and three months prior to his presentation, respectively. He had a negative stool O&P in 2017 but never had serological testing for *Strongyloides* previously.

We determined that he had *Strongyloides* hyperinfection syndrome (SHS) with likely disseminated strongyloidiasis exacerbated by dexamethasone for COVID-19 disease. He was initiated on ivermectin 200 mcg/kg/day initially via nasogastric tube and then subcutaneous injection once available (obtained through a local veterinary pharmacy) given the possibility of suboptimal oral ivermectin absorption. Albendazole 400 mg PO BID was added and continued with ceftriaxone for empiric pneumonia treatment. After much discussion among various experts, in attempting to balance the expected benefit of using corticosteroids to treat COVID-19 disease and the potential harm of prolonging disseminated strongyloidiasis, it was ultimately decided to continue with dexamethasone.

Four days later, the patient became febrile to 39.4℃ (102.9 °F) with worsening hypoxemia and hypotension. He developed anuric acute kidney injury requiring continuous renal replacement therapy. His blood cultures demonstrated Gram-positive and Gram-negative bacilli, (admission blood cultures were negative) and his tracheal aspirate culture demonstrated Gram-negative bacilli; thus, ceftriaxone was stopped and piperacillin-tazobactam and vancomycin were started. Given the patient’s clinical deterioration, his family decided to transition him to comfort care and he passed away that day.

His blood cultures subsequently grew *Bifidobacterium* species and extended-spectrum b-lactamase *Klebsiella oxytoca*. His tracheal aspirate grew coliforms. His sputum O&P was negative for larvae. He had no source of infection other than the GI tract.

## Discussion

Untreated chronic strongyloidiasis can predispose to *Strongyloides* hyperinfection syndrome (SHS) and disseminated strongyloidiasis, especially in the setting of immunosuppression [8]. SHS occurs through a process of accelerated autoinfection of the parasite with an increased number of larvae in the gastrointestinal tract and lungs. With disseminated strongyloidiasis, there is subsequent migration of larvae to organs outside the typical pulmonary autoinfection cycle, such as the liver, heart, and brain [9]. A common complication of this is Gram-negative bacteraemia secondary to translocation of enteric bacteria accompanying larval invasion of the gut wall [6]. SHS confers a mortality rate of greater than 85% [10].

Corticosteroid use is the most important risk factor for development of SHS in high-income countries [8]. This association is hypothesized to be through the acute suppression of cytokines such as IL-5, eosinophil and lymphocyte proliferation and activation, and by directly increasing the fertility of adult female worms [11]. This risk is independent of the duration, dose, and route of corticosteroid administration [8]. Courses of corticosteroids as short as six days and with a dose of oral prednisone as low as 20 mg per day have been shown to result in SHS [6]. Other immunomodulating medications such as methotrexate have also demonstrated association with accelerating Strongyloides’ autoinfection cycle [12]. In our case, the pre-existing immunosuppression for rheumatoid arthritis with methotrexate, hydroxychloroquine, and sulfasalazine likely contributed to his symptoms leading up to presentation and primed his condition for the rapid progression of larvae dissemination after receiving corticosteroids.

Our case highlights the diagnostic and management challenges of strongyloidiasis and the importance of epidemiologic history in determining risk. Patients often present with non-specific symptoms, which may lead to diagnostic and treatment delays. Furthermore, stool examination for parasites detects less than 30% of the cases given irregular shedding, with higher sensitivities during increased parasite burden and excretion [13]. Serologic testing is the most sensitive diagnostic test, but false negatives occur frequently. False negatives occur even more commonly in patients on immunosuppression. False positives can also occur with other helminth infections.

Epidemiologic risk factors for strongyloidiasis include birth, residence in, or travel to Sub-Saharan Africa, South America, the Caribbean, Southeast Asia, and Oceania. Even though strongyloidiasis is endemic to these tropical and subtropical regions, there is increasing prevalence in traditionally non-endemic countries due to travel and migration. Infection may occur with even very short periods of exposure, especially in people residing in rural settings or frequenting beach areas [10, 14]. There are several case reports describing strongyloidiasis acquired by short-term travellers, even as short as a week, and clinicians must assess this epidemiologic risk [14, 15]. Also, because strongyloidiasis is a lifelong infection until treated, any travel, regardless of remoteness, is relevant for risk assessment [14–16] Given the high mortality associated with SHS, clinicians must have a high index of suspicion for untreated chronic strongyloidiasis in any patient with epidemiologic risk. A combination of risk assessment along with serologic and stool testing is needed prior to starting immunosuppression [13].

Universal serologic testing for *S. stercoralis* in patients with COVID-19 who have epidemiologic risk has been recommended by some authors [17]. However, there are limitations to serological testing as the sensitivity of serology is lower in those who are immunocompromised or on immunosuppressive medications [18]. Empiric treatment with ivermectin in at-risk patients is a reasonable strategy due to the delayed turn-around time for serologic testing and the time-sensitive nature of starting corticosteroid therapy for COVID-19 disease [19]. A similar strategy has been used previously in at-risk patients undergoing immunosuppression, as research demonstrates that the cost-effectiveness of empiric treatment is non-inferior to a “test and treat” approach [10]. Another strategy is to initiate early treatment for strongyloidiasis in new refugees/migrants at the time of migration/arrival, but policies and practices vary in different geographic locations and clinicians should not rely on the assumption that Strongyloides risk has been addressed on migration/arrival and must do their own individual risk assessment for Strongyloides, especially if initiating any immunosuppressive medications [20].

Clinical risk factors associated with progression of strongyloidiasis and development of SHS include HTLV-1/2 infection, treatment with glucocorticoid or immunomodulatory agents, organ transplantation and hematologic malignancies [10]. The presence of unexplained serum eosinophilia in a patient who was born in or has travelled to an endemic region should also prompt investigation for strongyloidiasis. Peripheral eosinophilia, however, is not a sensitive marker for *Strongyloides* infection, and therefore its absence does not rule out infection [13].

Given the risk of SHS, consideration should be given for treating those at high risk of strongyloidiasis empirically if they will be initiated on corticosteroids or other immunosuppressives [10, 21]. While there have been attempts to study the optimal dosing of oral Ivermectin, the studies have been underpowered, have not directly studied all dosing regimens, or have a large number of participants lost to follow up [21, 22]. Two doses of oral Ivermectin 200 ug/kg/d usually separated by 2 weeks is the current practice standard. For people who are already immunosuppressed, additional doses of Ivermectin may be required. Additionally, albendazole 400 mg orally twice daily should be considered in patients with suspected SHS or disseminated strongyloidiasis, as well as empiric antibiotics with activity against gram-negative bacteria [23]. For those unable to tolerate ivermectin orally or suspected to have decreased gut absorption, it should be administered subcutaneously or intramuscularly [24]. In patients from West or Central Africa, special attention must be made to prevent the precipitation of ivermectin associated encephalopathy in the context of concurrent untreated loiasis [10].

## Conclusion

The use of immunosuppressive medications in clinical practice generally continues to increase and is standard of practice in the treatment of COVID-19. Our case highlights the importance of assessing for Strongyloides when using immunosuppressive medications for patients who have risk factors. Clinicians must do their own individual risk assessment for Strongyloides and not rely on refugee/migration policy. Screening may be difficult to undertake in the context of rapidly progressive disease or disease needing imminent initiation of immunosuppressive medication, therefore, strong consideration should be given to empirically treating those at high risk for disseminated strongyloidiasis or presenting with compatible illness. Also, more research highlighting the burden of strongyloidiasis and other opportunistic infections in COVID-19 patients needs to be undertaken.

## Data Availability

Consultation documents pertaining to the case are available upon request.
